# Green Voice Behavior in Nursing: The Roles of Organizational Support, Intrinsic Motivation, and Psychological Safety

**DOI:** 10.1155/jonm/1016556

**Published:** 2026-08-12

**Authors:** Seung Eun Lee, Heejin Lim, Sujin Nam, V. Susan Dahinten

**Affiliations:** ^1^ College of Nursing, Mo-Im KIM Nursing Research Institute, Yonsei University, Seoul, South Korea, yonsei.ac.kr; ^2^ College of Nursing, Yonsei University, Seoul, South Korea, yonsei.ac.kr; ^3^ School of Nursing, University of British Columbia, Vancouver, Canada, ubc.ca; ^4^ Faculty of Nursing, University of Tabuk, Tabuk, Saudi Arabia, ut.edu.sa

**Keywords:** green behavior, health facilities, nurses, proactive behavior, speaking up, sustainability, voice behavior

## Abstract

**Background:**

Coordinated action is needed to combat climate change and environmental damage, and it is important that healthcare be part of that response. Through proactive behaviors such as environmentally responsible communication or “green voice behavior,” nurses can effectively support sustainability initiatives. However, empirical evidence on the factors that encourage such behavior remains limited. This study examined the relationship between perceived green organizational support and nurses’ green voice behavior, while also testing the mediating role of green intrinsic motivation and the moderating effect of psychological safety.

**Design:**

A descriptive correlational design with cross‐sectional data was employed.

**Methods:**

Data were collected from 649 nurses in South Korea. We tested a second‐stage moderated mediation in which perceived green organizational support is associated with green voice behavior through green intrinsic motivation, while psychological safety moderates the association between green intrinsic motivation and green voice behavior. Analyses were conducted using the PROCESS macro in SPSS (Model 4 for simple mediation; Model 14 for moderated mediation).

**Results:**

Perceived green organizational support was positively associated with green voice behavior, both directly (*b* = 0.40, 95% confidence interval [CI] [0.329, 0.479]) and via green intrinsic motivation (*b* = 0.05, 95% CI [0.023, 0.075]). In our model, psychological safety moderated the relationship between green intrinsic motivation and green voice behavior (interaction *b* = 0.19, *p* = 0.002). The conditional indirect effect of perceived green organizational support on green voice behavior via green intrinsic motivation increased as psychological safety rose (index of moderated mediation = 0.03, 95% CI [0.010, 0.053]).

**Conclusion:**

These findings highlight the importance of supportive organizational environments in encouraging nurses to engage in environmentally responsible communication. Recognizing nurses’ environmental contributions, fostering intrinsic motivation, and ensuring psychological safety may promote green voice behavior in healthcare.

## 1. Introduction

Climate change and environmental degradation are global problems that call for coordinated action across all sectors. The healthcare industry is a key part of this effort, but its resource‐intensive operations create a significant environmental burden [1]. In this context, nurses are uniquely positioned to promote environmentally responsible healthcare as they are involved in nearly every aspect of clinical practice and work closely with patients and interdisciplinary teams [2]. To make a real difference, nurses may need to go beyond routine duties and speak up with suggestions or concerns about unsustainable practices.

*Green voice behavior* is a type of employee voice that focuses on environmental issues at work [3, 4]. It involves discretionary expressions of concern, suggestions, or ideas aimed at promoting sustainable practices or challenging environmentally harmful actions [5]. Unlike general voice, which covers broader organizational topics [6], green voice behavior reflects a strong personal commitment to environmental values [5] and often involves taking interpersonal risks to support organizational sustainability [7]. These risks are heightened in healthcare, where hierarchical organizations may inhibit individuals from voicing concerns [8]. Additional features, such as protocol‐driven and highly regulated care [9], continuous operations with heavy workloads [10], and multidisciplinary hierarchies [11], make speaking up even harder. Moreover, demands for patient safety and infection prevention may seem to be at odds with efforts to reduce waste, which may make nurses hesitant to voice their concerns or suggest other options [12]. Although green voice behavior has drawn attention in sectors such as oil and gas [13] and hospitality [7], its antecedents in healthcare remain underexplored, underscoring the need to identify factors that encourage nurses’ green voice behavior.

According to organizational support theory (OST) [14], employees are more inclined to put in more effort, including extra‐role behaviors, when they feel that their contributions are recognized and their well‐being is important to the organization [15, 16]. Extending OST to the environmental domain, green organizational support reflects employees’ perceptions that the organization values and supports employees’ environmental contributions [17, 18]. Studies conducted outside the healthcare sector consistently associate this perception with an increased willingness to speak up about sustainability. For example, employees who perceive strong green organizational support proactively share sustainability‐related information and experiences with coworkers [19] and propose novel ideas or changes to counter environmentally harmful practices [17]. Based on this prior evidence, it is reasonable to hypothesize that nurses who perceive stronger green organizational support are more likely to voice ideas that promote sustainability.

**Hypothesis 1.**

Perceived green organizational support is positively associated with green voice behavior.

When nurses feel that their hospital supports their work—that it values their contributions and backs efforts to improve practices—the work is more likely to feel meaningful and satisfying, and it tends to create more intrinsic interest [16]. According to OST, perceived support fulfills socioemotional needs and fosters positive affect and identity, which function as motivational mechanisms that enhance intrinsic interest in the supported work area [14]. In our context, the perception that environmental efforts are appreciated and supported offers a credible foundation for stronger green intrinsic motivation [20].

**Hypothesis 2.**

Perceived green organizational support is positively associated with green intrinsic motivation.

Green intrinsic motivation—an internal desire to act sustainably because such action is interesting, satisfying, and aligned with personal values—has been associated with discretionary green behaviors [21]. Unlike extrinsic incentives, this type of motivation reflects the inherent satisfaction derived from environmental sustainability actions [22, 23]. Individuals with strong green intrinsic motivation are more likely to suggest proenvironmental practices or raise concerns about harmful behaviors, even in the absence of formal rewards [21]. Empirical studies have linked green intrinsic motivation to a range of discretionary green behaviors, including green creativity [23] and proactive environmental action [22]. Because green voice behavior is discretionary and may involve interpersonal risk, nurses are more likely to express sustainability‐related suggestions or concerns when they are intrinsically motivated to support environmental sustainability.

**Hypothesis 3.**

Green intrinsic motivation is positively associated with green voice behavior.

In the broader voice literature, voice is understood as discretionary and self‐initiated rather than dictated by role [24]. Green voice behavior shares this discretionary and socially risky nature [13], which implies the need for a strong internal drive. When nurses have a higher level of green intrinsic motivation, they are more willing to initiate, persist in, and articulate sustainability‐related ideas despite potential interpersonal costs. From an OST perspective [14], perceiving higher green organizational support signals that the organization values and supports environmental efforts, which is associated with greater intrinsic interest and engagement in proenvironmental behavior. Taken together, higher perceived green organizational support is expected to lead to higher green intrinsic motivation and, in turn, greater green voice behavior.

**Hypothesis 4.**

Green intrinsic motivation mediates the relationship between perceived green organizational support and green voice behavior.

Although intrinsic motivation is essential for employee voice, it may not be sufficient in contexts where speaking up entails interpersonal risk. Psychological safety—a belief that one can express ideas, concerns, and suggestions without fear of negative consequences [25]—is widely identified as a near‐necessary condition for voice [24]. In hospitals, formal authority gradients and seniority norms can make open communication feel risky even for highly motivated staff [26]. Accordingly, we expect psychological safety to strengthen the association between green intrinsic motivation and green voice behavior. If psychological safety amplifies the relationship between green intrinsic motivation and green voice behavior, then the indirect effect of perceived green organizational support on green voice behavior via green intrinsic motivation will be stronger when psychological safety is high.

**Hypothesis 5.**

Psychological safety moderates the relationship between green intrinsic motivation and green voice behavior, such that the relationship is stronger when psychological safety is high.

**Hypothesis 6.**

The indirect effect of perceived green organizational support on green voice behavior through green intrinsic motivation is stronger when psychological safety is high.

**FIGURE 1:**
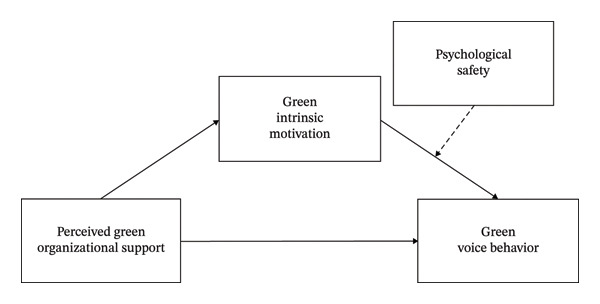
Hypothesized second‐stage moderated mediation model. Perceived green organizational support is associated with green voice behavior through green intrinsic motivation. Psychological safety moderates the relationship between green intrinsic motivation and green voice behavior, thereby strengthening the indirect effect of perceived green organizational support on green voice behavior. The dashed line indicates the moderating effect of psychological safety.

Taken together, the proposed relationships form a second‐stage moderated mediation model, as illustrated in Figure 1. This study contributes to the literature in several ways. First, it extends research on green voice behavior to the healthcare context, where hierarchical structures, professional norms, and patient safety priorities may uniquely constrain employees’ willingness to speak up about environmental sustainability. Second, it distinguishes green voice behavior from general voice behavior by emphasizing its sustainability‐oriented and value‐driven nature, which may create unique tensions in healthcare settings where environmental goals can conflict with infection control and safety demands. Finally, this study applies OST [14] to explain how perceived green organizational support is associated with green intrinsic motivation and, in turn, green voice behavior, while also identifying psychological safety as a contextual condition that strengthens this relationship.

## 2. Methods

### 2.1. Participants and Procedure

This correlational study utilized survey data collected from registered nurses employed at six hospitals in South Korea and was reported in accordance with the Strengthening the Reporting of Observational Studies in Epidemiology (STROBE) statement (available here). We collected data between January and February 2025 using convenience sampling. Eligible participants were registered nurses with at least 6 months of postlicensure clinical experience. To keep the focus on staff‐level perceptions, we excluded individuals in formal managerial roles. An online survey link was distributed by designated hospital contacts, and nurses were invited to share the link with qualified colleagues within their hospitals. Because of this open recruitment process, we could not determine the response rate. In total, 650 nurses completed the questionnaire; we removed one straight‐lining case (identical responses throughout), yielding 649 analyzable cases. The final sample size was sufficient to detect small effect sizes while ensuring a statistical power of 0.8 in mediation analysis using bias‐corrected bootstrapping [27].

### 2.2. Measurements

#### 2.2.1. Dependent Variable

Green voice behavior was assessed using a three‐item scale developed by Aboramadan et al. [28]. An example item is, “I speak up and encourage others to get involved in issues that affect the environment.” Participants rated their level of agreement on a five‐point Likert scale, ranging from 1 (*strongly disagree*) to 5 (*strongly agree*), with higher scores indicating greater engagement in green voice behavior. Cronbach’s alpha was reported as 0.92 in the previous study [28] and 0.89 in the present study.

#### 2.2.2. Independent Variable

Perceived green organizational support was assessed using a four‐item scale developed by Pinzone et al. [29]. An example item is, “The organization values my contribution to environmental management.” Responses were measured on a five‐point Likert scale, ranging from 1 (*strongly disagree*) to 5 (*strongly agree*), with higher scores indicating greater perceived green organizational support. Cronbach’s alpha was 0.80 in Pinzone et al.’s study [29] and 0.93 in the present study.

#### 2.2.3. Mediating Variable

Green intrinsic motivation was measured using a three‐item scale developed by Kim and Park [30]. A sample item is, “I feel good about my environmentally friendly behavior.” Responses were recorded on a five‐point Likert scale ranging from 1 (*strongly disagree*) to 5 (*strongly agree*), with higher scores indicating a greater level of intrinsic motivation toward environmentally sustainable behavior. Cronbach’s alpha was 0.86 in Kim and Park’s study [30] and 0.82 in the present study.

#### 2.2.4. Moderating Variable

In line with a previous study [31], psychological safety was measured using four items from Edmondson’s Psychological Safety Scale [25]. An example item is, “It is safe to take a risk on this team.” Responses were rated on a five‐point Likert scale, ranging from 1 (*strongly disagree*) to 5 (*strongly agree*), with higher scores indicating greater perceived psychological safety. Cronbach’s alpha was 0.67 in the prior study [31] and 0.76 in the present study.

### 2.3. Statistical Analysis

All analyses were conducted using SPSS Statistics 28.0 and PROCESS macro Version 5.0. Descriptive statistics summarized demographic characteristics and key study variables. Pearson’s bivariate correlations assessed associations between study variables. Following Hayes [32] and the stepwise procedure outlined by Jung and Seo [33], we estimated the hypothesized moderated mediation model using regression. First, we tested the simple mediation of green intrinsic motivation in the relationship between perceived green organizational support and green voice behavior with PROCESS Model 4 [34]. We then tested a second‐stage moderated mediation (PROCESS Model 14) in which psychological safety moderates the association between green intrinsic motivation and green voice behavior, rendering the indirect effect of perceived green organizational support conditional on psychological safety [34]. To facilitate the interpretation of an interaction term, all variables were mean‐centered prior to analysis [35]. We probed the moderation using simple slopes at the mean and at one standard deviation above and below the mean of psychological safety [32, 36]. Mediation and moderated mediation were examined with a bias‐corrected bootstrapping procedure based on 10,000 resamples and 95% confidence intervals (CIs) [32]. All regression models controlled for variables previously shown to be linked to voice behavior, including sex, age, educational level [7], hospital type [8], and unit tenure [37].

### 2.4. Ethics Statement

Prior to participating in this study, all participants were informed that their involvement was voluntary and that they had the right to withdraw at any time without any consequences. They were also assured of the confidentiality and secure handling of their survey responses. All procedures were conducted in accordance with the ethical standards outlined in the Declaration of Helsinki. Ethical approval for this study was obtained from the Institutional Review Board of Yonsei University Health System (4‐2025‐0485).

## 3. Results

### 3.1. Demographic Characteristics

As shown in Table 1, the majority of participants were female (95.7%), with a mean age of 33.66 (SD = 7.49) years. Nearly all held a bachelor’s degree or higher (95.5%) and were employed at tertiary care hospitals (84.9%). The participants had an average tenure of 5.39 (SD = 5.31) years in the unit and 9.64 (SD = 7.48) years in the hospital.

**TABLE 1:** Demographic characteristics of the sample (*N* = 649).

Characteristic	Category	*n* (%)	Mean ± SD
Sex	Female	621 (95.7)	
	Male	28 (4.3)	

Age (years)			33.66 ± 7.49

Educational level	Associate degree	29 (4.5)	
	Bachelor’s degree or higher	620 (95.5)	

Tenure in the unit (years)			5.39 ± 5.31

Tenure in hospital (years)			9.64 ± 7.48

Hospital type	Tertiary hospital	551 (84.9)	
	General hospital	98 (15.1)	

Abbreviation: SD = standard deviation.

### 3.2. Descriptive Statistics and Bivariate Correlations Among the Key Variables

As presented in Table 2, perceived green organizational support showed a significant positive correlation with green intrinsic motivation (*r* = 0.20, *p* < 0.001), psychological safety (*r* = 0.31, *p* < 0.001), and green voice behavior (*r* = 0.42, *p* < 0.001). Green intrinsic motivation was positively correlated with both psychological safety (*r* = 0.09, *p* = 0.016) and green voice behavior (*r* = 0.32, *p* < 0.001). A positive relationship was found between psychological safety and green voice behavior (*r* = 0.13, *p* = 0.001).

**TABLE 2:** Descriptive statistics and Pearson’s bivariate correlation analyses (*N* = 649).

Variables	Mean ± SD	1	2	3
1. Perceived green organizational support	2.81 ± 0.76	—		
2. Green intrinsic motivation	3.89 ± 0.61	0.20^∗∗∗^	—	
3. Psychological safety	3.37 ± 0.64	0.31^∗∗∗^	0.09^∗^	—
4. Green voice behavior	2.58 ± 0.83	0.42^∗∗∗^	0.32^∗∗∗^	0.13^∗∗^

Abbreviation: SD = standard deviation.

^∗^ *p* < 0.05.

^∗∗^ *p* < 0.01.

^∗∗∗^ *p* < 0.001.

### 3.3. Mediation Tests (Hypotheses 1–4)

Before testing the full moderated mediation model, we examined the simple mediation effect of green intrinsic motivation on the association between perceived green organizational support and green voice behavior. Perceived green organizational support was positively associated with green intrinsic motivation (*b* = 0.15, *p* < 0.001), supporting Hypothesis 2. Green intrinsic motivation was positively associated with green voice behavior (*b* = 0.31, *p* < 0.001), supporting Hypothesis 3. As shown in Table 3, the indirect association of perceived green organizational support with green voice behavior via green intrinsic motivation was statistically significant (*b* = 0.05, 95% CI [0.023, 0.075]), supporting Hypothesis 4. The direct association between perceived green organizational support and green voice behavior remained statistically significant after accounting for green intrinsic motivation (*b* = 0.40, 95% CI [0.329, 0.479]), supporting Hypothesis 1 and indicating partial mediation.

**TABLE 3:** Total, indirect, and direct associations between perceived green organizational support and green voice behavior (*N* = 649).

Path	*b*	SE	95% CI (lower, upper)
Indirect association (via green intrinsic motivation)			
Perceived green organizational support ⟶ green voice behavior	0.05	0.01	0.023, 0.075
Direct association			
Perceived green organizational support ⟶ green voice behavior	0.40	0.04	0.329, 0.479

*Note: b* = unstandardized coefficient; 95% CIs are bias‐corrected bootstrap intervals (10,000 resamples).

Abbreviation: SE = standard error.

### 3.4. Moderation and Moderated Mediation Tests (Hypotheses 5‐6)

**FIGURE 2:**
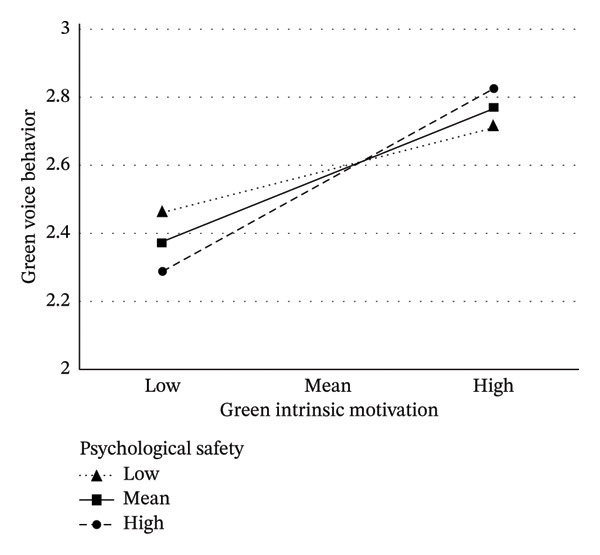
Interaction of green intrinsic motivation and psychological safety on green voice behavior. Slopes are plotted at −1 SD, the mean, and +1 SD of psychological safety.

Next, consistent with our second‐stage moderation hypothesis, we tested whether psychological safety moderates the association between green intrinsic motivation and green voice behavior. The interaction between green intrinsic motivation and psychological safety was significant (*b* = 0.19, *p* = 0.002), supporting Hypothesis 5; this indicates that individuals with higher psychological safety showed a stronger positive link between intrinsic motivation and voice. Simple slopes analysis (Figure 2) showed that this relationship was strongest at high psychological safety (*b* = 0.44, *p* < 0.001), followed by the mean (*b* = 0.32, *p* < 0.001) and then low levels of psychological safety (*b* = 0.20, *p* = 0.001).

Finally, we examined the moderated mediation model. The index of moderated mediation was statistically significant (index of moderated mediation = 0.03, 95% CI [0.010, 0.053]), supporting Hypothesis 6; this suggests that the conditional indirect association between perceived green organizational support and green voice behavior through green intrinsic motivation increased with psychological safety. As shown in Table 4, the conditional indirect relationships were significant at all levels of psychological safety, with the strongest association at high levels of psychological safety (*b* = 0.06, 95% CI [0.033, 0.104]), followed by the mean level (*b* = 0.05, 95% CI [0.024, 0.078]) and low levels of psychological safety (*b* = 0.03, 95% CI [0.011, 0.058]).

**TABLE 4:** Conditional indirect association between perceived green organizational support and green voice behavior via green intrinsic motivation at different levels of psychological safety (*N* = 649).

Level of psychological safety	*b*	SE	95% CI (lower, upper)
Low (–1 SD)	0.03	0.01	0.011, 0.058
Mean	0.05	0.01	0.024, 0.078
High (+1 SD)	0.06	0.02	0.033, 0.104

*Note: b* = unstandardized coefficient; CIs are bias‐corrected bootstrap intervals (10,000 resamples).

Abbreviations: SD = standard deviation, SE = standard error.

## 4. Discussion

This study examined how perceived green organizational support is related to nurses’ green voice behavior and the conditions under which this association is strengthened. Overall, the findings support all hypothesized relationships and provide insights into how organizational and psychological factors are associated with green voice behavior in healthcare settings.

In line with prior research conducted in nonhealthcare contexts such as hospitality [19] and engineering [13], we found a significant positive association between perceived green organizational support and green voice behavior, supporting Hypothesis 1. This finding aligns with OST, which suggests that employees who feel valued and supported by their organization are more inclined toward extra‐role behaviors [14, 16]. In the healthcare context, where sustainability efforts may compete with clinical priorities [12], perceived organizational support may help legitimize environmental concerns and signal that such efforts are valued. As a result, nurses may be more willing to express green voice behavior by offering suggestions, raising concerns, and proposing sustainability initiatives.

Consistent with Hypothesis 2, perceived green organizational support was positively associated with green intrinsic motivation among nurses. This relationship can be understood through the lens of OST, which suggests that perceiving one’s contributions as valued and supported signals organizational backing and fosters positive internal experience [14]. In the context of environmental efforts, such support may make sustainability‐related work feel more gratifying and self‐affirming, thereby enhancing intrinsic motivation [38, 39]. When nurses perceive that their environmental contributions are recognized and supported, they may be more likely to internalize these values and experience greater personal meaning in proenvironmental activities.

In line with Hypothesis 3, green intrinsic motivation was positively associated with green voice behavior. Nurses with stronger intrinsic motivation toward environmental sustainability were more likely to express sustainability‐related suggestions and concerns in the workplace. Because employee voice is typically characterized as discretionary [6], intrinsic motivation may play an important role in sustaining the effort required to engage in green voice behavior. This finding aligns with prior research suggesting that intrinsically motivated employees are more inclined to engage in discretionary behaviors, as such actions are experienced as inherently rewarding [13, 40]. In healthcare settings, nurses who view environmental sustainability as personally meaningful may be more willing to engage in green voice behavior despite potential interpersonal or organizational risks.

Supporting Hypothesis 4, green intrinsic motivation mediated the relationship between perceived green organizational support and green voice behavior. This finding suggests that perceived green organizational support may foster green voice behavior by strengthening nurses’ intrinsic motivation toward environmental sustainability. When nurses perceive that their environmental efforts are valued and supported, they are more likely to internalize sustainability‐related goals and view environmentally responsible practices as personally meaningful [14, 39]. This motivational state may, in turn, increase their willingness to express sustainability‐related suggestions and concerns despite the discretionary nature of voice behavior [13, 40]. By identifying green intrinsic motivation as an underlying mechanism, the present findings extend prior research on organizational support and intrinsic motivation [38, 39] to the environmental context of healthcare.

In support of Hypothesis 5, psychological safety strengthened the relationship between green intrinsic motivation and green voice behavior. Specifically, the positive association between green intrinsic motivation and green voice behavior was stronger when nurses perceived high levels of psychological safety. This finding suggests that even when nurses are intrinsically motivated, low psychological safety may constrain the expression of green voice behavior. Because employee voice is inherently interpersonal and entails the risk of disapproval or professional consequences [24], green voice behavior may be particularly sensitive to contextual conditions such as psychological safety. This boundary condition may be particularly important in healthcare settings, where hierarchical norms and rigid organizational structures can inhibit open communication [41, 42]. In such contexts, psychological safety may reduce the perceived risks associated with expressing green voice behavior [43] and signal respect for employees’ contributions [44], thereby enabling nurses to act on their intrinsic motivation.

In further support of the proposed model, psychological safety also moderated the indirect association between perceived green organizational support and green voice behavior through green intrinsic motivation. The indirect association was stronger at higher levels of psychological safety, suggesting that motivational and contextual factors jointly influence green voice behavior. Although organizational support may strengthen nurses’ intrinsic motivation toward environmental sustainability, the absence of psychological safety may still inhibit the expression of green voice behavior. This finding underscores the importance of aligning motivational and contextual resources, as motivated employees may withhold sustainability‐related ideas or concerns when they perceive interpersonal risk. By demonstrating this combined effect, the present study extends previous qualitative research suggesting that individual motivation, contextual enablers, and organizational factors are jointly associated with green voice behavior [13] and offers quantitative evidence for these relationships in the nursing context.

Although the observed effect sizes were modest, they remain meaningful in the context of healthcare settings, where behavioral change can be difficult due to hierarchical structures, heavy workloads [45], and competing organizational priorities [12]. In complex organizational environments such as hospitals, even relatively small effects may have practical implications when accumulated across employees and sustained over time. Thus, the present findings suggest that organizational and psychological factors associated with green voice behavior may still contribute meaningfully to promoting environmentally sustainable practices in healthcare settings.

### 4.1. Implications for Nursing Management

This study offers several practical implications for nursing management seeking to promote green voice behavior. First, organizations should demonstrate their commitment to environmental sustainability through formal recognition programs, by including nurses in environmental decision‐making (e.g., participation in sustainability committees), and keeping lines of communication open for feedback (e.g., regular team meetings or suggestion systems) [16, 40]. Targeted environmental education and eco‐literacy programs can further strengthen perceptions of organizational support [18, 46]. Second, nurse managers can help nurses internalize environmental values and find personal meaning in sustainability work by providing opportunities to lead or participate in green projects (e.g., unit‐level waste reduction or energy‐saving initiatives) [40], showing them clear evidence of the effects of their environmental actions [47], and acknowledging these contributions [47, 48]. These practices can help nurses build autonomy, competence, and connectedness. Finally, it is important to create psychologically safe environments that allow nurses to share their ideas and concerns about how environmental practices are done in the workplace. To foster psychological safety, leaders can model openness and respect for diverse perspectives (e.g., by explicitly inviting input during team discussions and responding constructively to suggestions), while hospitals can allocate resources toward leadership development that enable managers to cultivate inclusive, trust‐based teams [44]. Our findings suggest that visible organizational commitment, support for intrinsic motivation, and a psychologically safe environment can promote green voice behavior among nurses.

### 4.2. Study Limitations

This study has several limitations. First, all variables were measured using self‐reported data collected at a single time point, which raises the possibility of common method bias and limits causal inference [49]. Although the hypothesized relationships were theory‐driven, the observed associations may have been influenced by shared method variance. Future research could address this limitation by incorporating multisource data (e.g., supervisor or peer evaluations) or longitudinal designs. Second, the recruitment strategy, including participant‐driven sharing of the survey link, may have introduced selection bias by disproportionately attracting individuals with a greater interest in environmental sustainability. Even though the sample showed variability across key constructs, the possibility of such bias cannot be ruled out. Future studies should therefore include more diverse samples across healthcare settings, professional roles, and cultural contexts. Third, this study did not examine other factors that may influence green voice behavior, such as job embeddedness [4] or leadership styles [40]. Future research could incorporate these variables to provide a more comprehensive understanding of the antecedents of nurses’ green voice behavior. Finally, all key variables were measured using relatively short scales consisting of three or four items, which may have limited the breadth of construct representation. Despite the scales demonstrating acceptable internal consistency, future studies may benefit from using more comprehensive measures to further strengthen construct validity.

## 5. Conclusion

We examined the direct relationship between perceived organizational support and nurses’ green voice behavior, as well as the indirect relationship through green intrinsic motivation. We also investigated whether psychological safety had an impact on this indirect relationship. Our findings show that organizational support is related to nurses’ intrinsic motivation to engage in environmentally responsible actions. This, in turn, promotes their willingness to voice ideas, suggestions, and concerns related to sustainability, particularly in situations where psychological safety is high. It is possible to encourage nurses to voice concerns and share ideas about sustainability in healthcare settings by creating conditions that strengthen nurses’ intrinsic motivation and ensure psychological safety. Additional motivational and contextual factors could be investigated in future studies, as well as whether similar patterns occur in diverse healthcare settings and cultural contexts.

## Author Contributions

Conceptualization and supervision: Seung Eun Lee; methodology, investigation, and writing–original draft: Seung Eun Lee, Heejin Lim, and Sujin Nam; formal analysis: Heejin Lim; writing–review and editing: Seung Eun Lee, Heejin Lim, Sujin Nam, and V. Susan Dahinten.

## Funding

This research received no external funding.

## Conflicts of Interest

The authors declare no conflicts of interest.

## Supporting Information

Additional supporting information can be found online in the Supporting Information section.

## Supporting information


**Supporting Information** STROBE statement—checklist of items that should be included in reports of *cross-sectional studies*.

## Data Availability

The data that support the findings of this study are available on request from the corresponding author. The data are not publicly available due to privacy or ethical restrictions.
